# Horizontal stenting via retrograde route for recurrent ruptured posterior communicating artery aneurysm after clipping: A case report and literature review

**DOI:** 10.1002/ccr3.5920

**Published:** 2022-05-27

**Authors:** Michiyasu Fuga, Toshihide Tanaka, Rintaro Tachi, Ryo Nogami, Akihiko Teshigawara, Toshihiro Ishibashi, Yuzuru Hasegawa, Yuichi Murayama

**Affiliations:** ^1^ Department of Neurosurgery Jikei University School of Medicine Kashiwa Hospital Chiba Japan; ^2^ 12839 Department of Neurosurgery Jikei University School of Medicine Tokyo Japan

**Keywords:** acutely angled, anterior communicating artery, fetal variant posterior cerebral artery, recurrent aneurysm, stent‐assisted

## Abstract

Treatment of recurrent ruptured aneurysms incorporating a branch vessel arising from the dome is challenging. Here, we attempted horizontal stent‐assisted coil embolization via a retrograde route from the contralateral internal carotid artery to treat a small ruptured posterior communicating artery aneurysm incorporating a fetal variant posterior cerebral artery after clipping.

## Graphical abstract

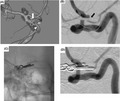

## 1 INTRODUCTION

Surgical treatment for recurrent aneurysms after clipping is considered cumbersome, especially for reclipping because of existing clips, scar tissue, and severe adhesions.^1^, ^2^ Therefore, to avoid additional surgical insult, coil embolization is considered advantageous compared with craniotomy surgery.^3^, ^4^ However, endovascular surgery for recurrent aneurysm incorporating a branch vessel arising from the dome is quite difficult. Stent‐assisted coiling (SAC) could overcome such difficulties compared with the standard technique. Various stent‐assisted techniques have been applied for SAC of bifurcated aneurysms.^5^, ^6^, ^7^ Horizontal stenting involves the placement of the stent across the aneurysm neck parallel to the bifurcated branch and axis of the neck, achieving optimal neck formation with only one stent.^8^ This procedure reduces the risk of thromboembolic complications and medical costs.^9^ In cases of stent deployment for a branch vessel acutely angled from the aneurysm sac, a retrograde approach via the anterior communicating artery (AcomA) allows easier guidance of the microcatheter than the anterograde approach,^9^, ^10^, ^11^ especially for retreatment after clipping. The technical limitations of this approach depend on the AcomA or posterior communicating artery (PcomA) diameter. The NeuroForm Atlas stent (Stryker, Kalamazoo, MI, USA) enables guidance to smaller blood vessels and is easier than guidance via microcatheter since the outer diameter in only 1.7 F. Here, we describe a case of ruptured small PcomA aneurysm incorporating a fetal variant posterior cerebral artery (PCA) after clipping, treated by horizontal stenting using a NeuroForm Atlas stent delivered via AcomA from the contralateral internal carotid artery (ICA).

## 2 CASE PRESENTATION

**FIGURE 1:**
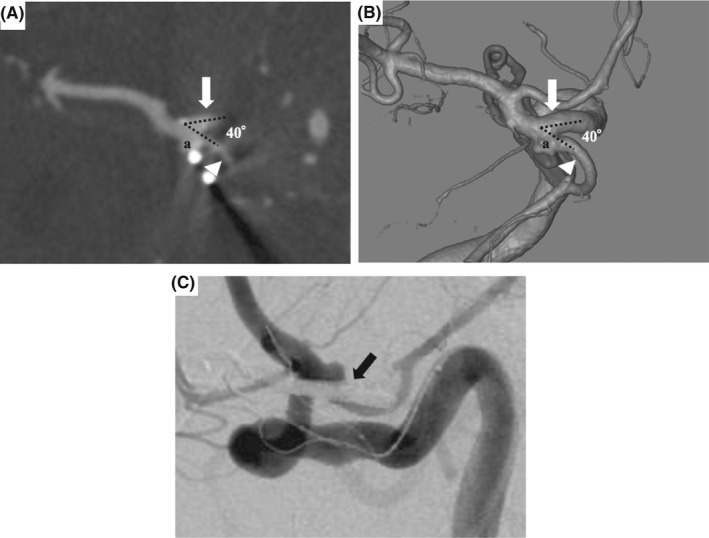
Axial computed tomography angiography source images (A) and 3‐dimensional digital subtraction angiography (image from directly above) (B) demonstrating a 2.7‐mm broad‐necked posterior communicating artery (PcomA) aneurysm residuum (a) just proximal to the clip incorporating an acute angled fetal variant posterior cerebral artery. These images show an acute angle (40°) subtended by the PcomA (white arrowhead) and proximal internal carotid artery (white arrow). The orifice of the PcomA is not well visualized on right internal carotid artery angiography (C) due to the existing clip

An 80‐year‐old woman who had underwent clipping surgery for a ruptured PcomA aneurysm 10 years earlier at another hospital developed subarachnoid hemorrhage (Hunt & Hess grade 2; Fisher group 3). Computed tomography angiography (CTA) and 3‐dimensional digital subtraction angiography demonstrated a residual PcomA aneurysm with a 2.7‐mm wide‐necked aneurysm just proximal to the clip and an isolated fetal variant PCA originating from the aneurysm sac (Figure 1A,B). The Allcock test (vertebral angiography accompanied by right carotid artery compression) showed completely absent right pre‐communicating segment (P1). Based on these findings, rupture of a residual PcomA aneurysm was diagnosed.

### 2.1 Treatment and technique

**FIGURE 2:**
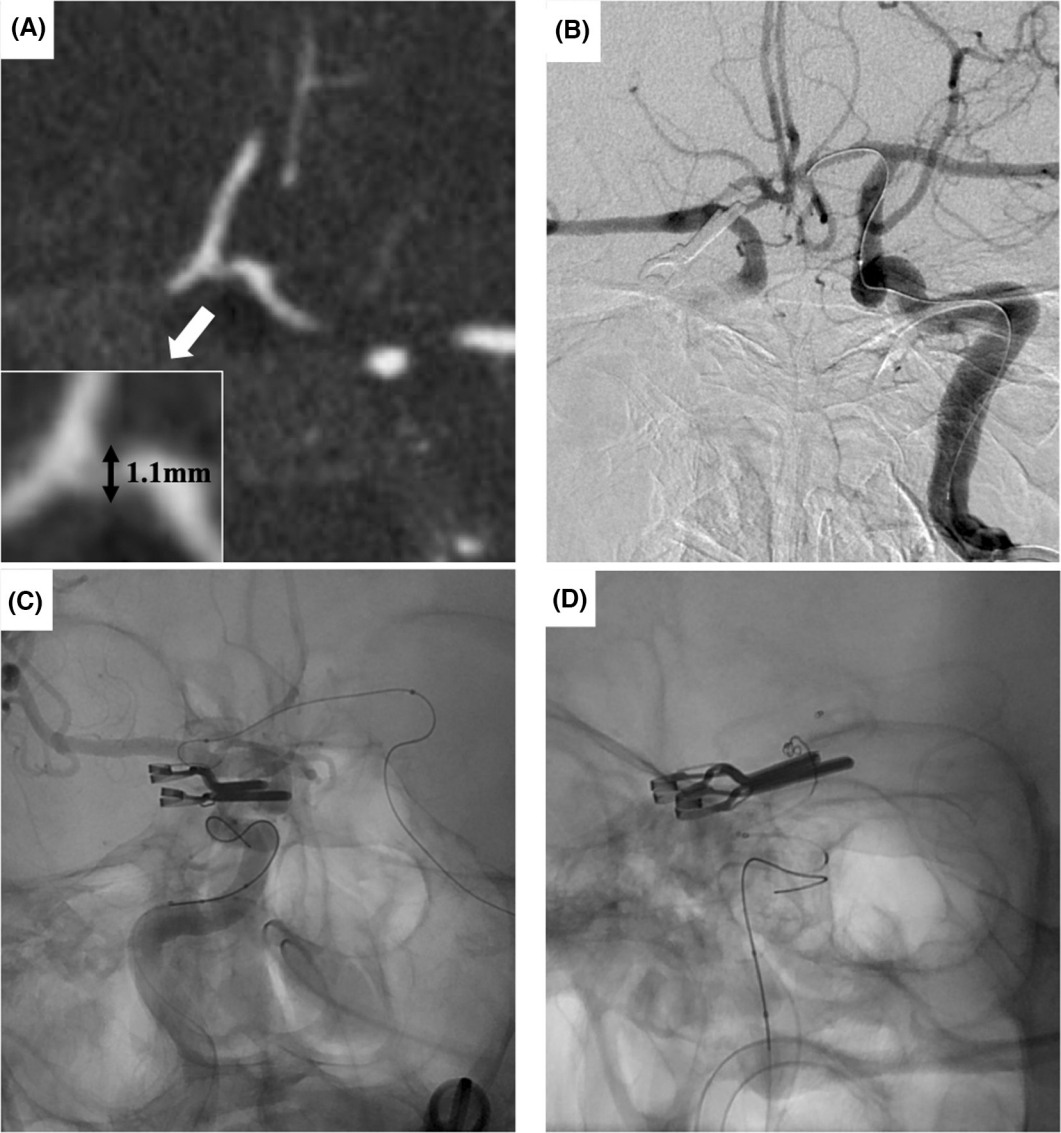
Axial computed tomography angiography source images (A) show anterior communicating artery with a diameter of 1.1 mm. Left internal carotid artery (ICA) angiography (B) under balloon inflation in the right proximal ICA and the fluoroscopic view (C and D) show retrograde navigation of the stent delivery catheter to the contralateral horizontal segment of the anterior cerebral artery (B), ipsilateral terminal ICA (C), and right fetal variant posterior cerebral artery (D) from the contralateral ICA.

**FIGURE 3:**
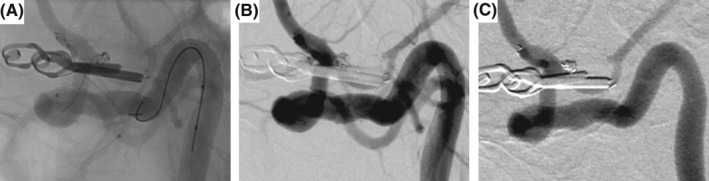
(A) Fluoroscopic view shows the NeuroForm Atlas stent deployed from the right fetal variant posterior cerebral artery to the terminal right internal carotid artery (ICA). (B) Right ICA angiography shows complete occlusion of the aneurysm without coil migration into the parent arteries. (C) Right ICA angiography at the two‐year follow‐up after endovascular treatment demonstrates complete occlusion without recanalization or in‐stent stenosis.

Because the residual aneurysm was small with a wide neck, and the fetal variant PCA branch vessel was arising from the dome, SAC was selected to preserve the PcomA. The stent was used with the consent of the patient and their family due to the off‐label use for ruptured aneurysm in Japan. Dual antiplatelet agents (loading doses: 200 mg aspirin, 200 mg cilostazol) were administered continuously before and after embolization. Endovascular treatment was performed under general anesthesia, and heparin was administered systemically as an initial 3000‐U bolus, followed by 1000 U/h with monitoring of whole‐blood activated clotting time throughout the procedure. An 8‐Fr FlowGate2 balloon guide catheter (Stryker) was guided to the petrous segment of the right ICA via the right femoral artery. An Excelsior SL‐10 microcatheter (Stryker) was guided into the aneurysm from the 8‐Fr FlowGate2 placed in the right ICA. After that, a Target 360 Nano coil (Stryker) was inserted into the aneurysm. However, because the aneurysm was small and wide‐necked, and the fetal variant PCA branch vessel was arising from the dome, placing the coil into the aneurysm alone so as to avoid herniating the ICA trunk and PcomA was difficult. The PcomA branched at an acute angle (40°) (Figure 1A,B) and the orifice of the PcomA was not well visualized due to the existing clip (Figure 1C). The microguide wire and the stent delivery microcatheter were shaped to fit into the orifice of the PcomA, but they could not be guided in an anterograde manner to the PcomA from the ipsilateral ICA. A 5‐Fr Envoy (Codman Neurovascular) was guided to the cervical segment of the contralateral ICA via the left femoral artery. Because the AcomA was 1.1 mm in size (Figure 2A), the stent delivery catheter was navigated in a retrograde manner to the contralateral horizontal segment of the anterior cerebral artery (A1) (Figure 2B), ipsilateral terminal ICA (Figure 2C), and right fetal variant PCA (Figure 2D) from the contralateral ICA via the AcomA. In the process of guiding the catheter from the contralateral ICA to the ipsilateral ICA across the AcomA, the microcatheter was mistakenly placed into the artery of Heubner. After guiding the stent delivery catheter to the right fetal variant PCA, a microcatheter for coil delivery via the 8‐Fr FlowGate2 placed at the ipsilateral ICA was advanced into the aneurysm sac. The self‐expandable 4.0 × 21 mm NeuroForm Atlas stent was navigated through the Excelsior SL‐10 placed in the right PcomA and deployed from the right PcomA into the terminal right ICA (Figure 3A). The coil delivery microcatheter was jailed. The aneurysm was completely occluded by three Target 360 Nano coils using the jailed microcatheter (Figure 3B). Final right internal carotid angiography (ICAG) demonstrated complete occlusion of the aneurysm by appropriate placement of the stent with preservation of the parent arteries, including the terminal right ICA and PcomA. After embolization, the patient suffered left hemiparesis. Postoperative magnetic resonance imaging showed cerebral infarction in the caudate head and internal capsule consistent with the territory of the right artery of Heubner. Dual antiplatelet therapy has been continued for three months. The patient was transferred to a rehabilitation hospital with a modified Rankin scale score of 4 as of two months after endovascular treatment. Follow‐up right ICAG two years after embolization demonstrated persistent complete occlusion without recanalization (Figure 3C).

## 3 DISCUSSION

The difficulties and pitfalls in the present case primarily involved the small, recurrent ruptured aneurysm. Reclipping for recurrent aneurysms following surgical clipping tends to be cumbersome, because the direction of insertion for the clip blade is restricted by existing clips, scar tissue, and adhesions.^1^, ^2^ In addition, before removing the previous clip, bypass is sometimes required to preserve blood flow of the efferent artery and to protect against ischemia caused by the prolonged intraoperative proximal temporary occlusion of the parent artery.^12^ On the other hand, Rabinstein et al. reported on 21 postsurgical neck remnants of aneurysms treated by endovascular coiling without major complications.^3^ Subarachnoid hemorrhage and symptomatic aneurysmal regrowth were not observed after endovascular retreatment during follow‐up (mean, 22 months).^3^ In good grade elderly patients with small ruptured anterior circulation aneurysms, coil embolization considered more suitable than clipping in terms of a shorter length of stay in the hospital, less infectious and pulmonary complications, and a lower frequency of epilepsy.^13^ For these reasons, coil embolization is more advantageous for recurrent ruptured aneurysm following clipping.^4^

In endovascular treatment, if the aneurysm is small, has a wide neck, and normal branch vessels arising from the dome, treatment with a simple technique is difficult. In the present patient, the digital subtraction angiography did not reveal P1 in the Allcock test, which means the sacrifice of PcomA is at high risk for ischemic complications.^14^ Therefore, to preserve the PcomA, some adjunctive techniques are required, such as balloon‐assisted technique (BAT), double‐catheter technique (DCT), and SAC. Abdalkader et al. pointed out that the balloon deflation phase during BAT for small aneurysms might increase the risk of coil protrusion and coil migration.^15^ When DCT is applied for small and ruptured aneurysms, placing two microcatheters in the aneurysm sac while avoiding perforation is difficult.^16^ Based on such description, SAC is considered a more appropriate treatment. Two options are available for SAC to treat bifurcation aneurysms, using the antegrade or retrograde route. Y‐stenting is useful for access via the antegrade route.^5^, ^17^ However, Y‐stenting requires two stents, which might increase the risk of thrombotic complications.^8^, ^18^ Johnson et al. pointed out that Y‐stenting represents an independent risk factor for permanent disabling neurological complications.^19^ Moreover, new neurointerventional devices such as flow diverter stents, the Woven EndoBridge (WEB) device (MicroVention, Aliso Viejo, CA, USA), and the PulseRider device (Cerenovus, Johnson & Johnson Medical Devices, New Brunswick, NJ, USA) have recently become available for wide neck cerebral aneurysms.^20^, ^21^, ^22^ However, flow diverter placement is not effective in preventing early rebleeding,^23^ and with regard to the WEB, no device is available for aneurysms smaller than 3 mm.^24^ Furthermore, appropriately implanting the PulseRider into the PcomA acutely angled from the aneurysm dome is quite difficult.^25^

The technical difficulties in this case involved the acute angle between the parent artery and PcomA. Guiding the stent delivery catheter into a branched vessel with an acute angle is difficult. Moreover, the existing clip obscures the PcomA/ICA junction in cases of recurrent aneurysm after surgical clipping. Anterograde guidance of the microcatheter into the branch vessel acutely angled from the aneurysm sac, particularly one with an existing clip, seems disadvantageous, therefore a retrograde approach should be assumed in advance. In fact, in the present treatment, despite shaping the microguidewires and stent delivery microcatheter to fit into the orifice of the PcomA, an anterograde guidance to the PcomA was unsuccessful. In addition, the intra‐aneurysmal microcatheter looping technique could be considered as a method of guiding catheters into difficult‐to‐guide branches.^26^ However, small or ruptured aneurysms have a high risk of aneurismal rupture during procedure. Waffle cone technique enables embolization while preserving blood flow without navigating the microcatheter to acutely angled efferent vessels.^27^ However, this procedure leads to not only the potential risk of perforation, especially for small aneurysms but also high probability of aneurysm recurrence.^28^ Kim et al reported a case of the ruptured PcomA aneurysm incorporated with a fetal variant PCA, resulting in recanalization after treatment for endovascular waffle cone stent‐assisted coiling.^28^ The retrograde approach facilitates guidance of the microcatheter toward the target vessel, because the angle of bifurcation between the terminal ICA and PcomA becomes obtuse. In addition, this procedure facilitates horizontal stenting, which covers the neck horizontally along the wall of the efferent and afferent arteries with a single stent. This technique not only leads to tight packing to reduce recanalization, but also prevents thrombotic complications compared with Y‐stents.

According to previous reports, 15 aneurysms, including that in the present case, have been treated by horizontal stenting delivered in a retrograde manner via the AcomA from the contralateral ICA.^7^, ^9^, ^10^, ^28^, ^29^, ^30^, ^31^, ^32^, ^33^ As shown in Table 1, all PcomA aneurysms incorporated a fetal variant PCA. The aneurysm type was unruptured in 11 cases and ruptured in 3, including 4 recurrent aneurysms. No treatment for recurrence of aneurysm was seen after previous clipping, except in the present study. Most aneurysms were ≥5 mm in size and had a wide neck. Ahmed et al. reported that in the treatment of a ruptured 2‐mm carotid terminus/A1 aneurysm, a double Enterprize stent (Codman Neurovascular) construct was deployed horizontally across the aneurysm in “telescopic” fashion for inflow coverage.^32^

**TABLE 1:** Horizontal stenting by retrograde technique via anterior communicating artery

Case	Authors	Age (years)/sex	Aneurism location	Aneurism type	Symptom	Aneurism size (mm)	Dome size (mm)	Neck size (mm)	Dome/Neck ratio	Proximal vessel diameter (mm)	Distal vessel diameter (mm)	Bifurcation vessel angle (°)	AcomA size (mm)	Stent delivery microcatheter	Stent	Stent size (mm)	Antiplatelet therapy	Immediate aneurism occlusion	Follow‐up duration (month)	Recurrence	Complications
1	Benndorf et al., 2006^7^	58/M	L ICA terminus	Unruptured	Mild headache	NR	NR	NR	NR	NR	NR	NR	NR	Prowler Select Plus	Enterprise	4.5 × 22	CLP (75 mg), ASP (325 mg)	Near‐complete occlusion	NR	NR	None
2	Kelly et al., 2007^29^	61/F	L ICA terminus	Unruptured (partially thrombosed)	NR	11 × 6 × 7 (patent), 26 (entire lesion)	NR	NR	NR	NR	NR	NR	NR	None	Neuroform 3	4.0 × 5	CLP (75 mg), ASP (325 mg)	Near‐complete occlusion	NR	None	None
3	Kelly et al., 2007^29^	66/F	L ICA terminus	Unruptured (clipping failure)	Headache	9 × 5	NR	6	NR	NR	NR	NR	NR	None	Neuroform 3	4.0 × 20	CLP (75 mg), ASP (325 mg)	Near‐complete occlusion	NR	None	None
4	Siddiqui et al., 2009^30^	47/F	ICA terminus	Ruptured (staged treatment)	NR	15	2.5	5	0.5	1.25	1.8	NR	NR	Prowler Select Plus	Enterprise	22	CLP, ASP	Residual neck	12 (DSA)	None	Groyne haematoma, reversible alopecia
5	Siddiqui et al., 2009^30^	27/F	ICA terminus	Recurrence (post‐coil embolization)	NR	5	2	3	0.7	1.5	1.9	NR	NR	Prowler Select Plus	Enterprise	22	CLP, ASP	Complete occlusion	6 (DSA)	None	None
6	Puri et al., 2009^31^	49/F	BA top	Recurrence (post‐coil embolization), unruptured	Headache	NR	NR	NR	NR	NR	NR	NR	NR	Prowler Select Plus	Enterprise	4.5 × 22	CLP (75 mg), ASP (325 mg)	Complete occlusion	3 (MRA)	None	None
7	Albuquerque et al., 2011^9^	56/F	L ICA terminus	Unruptured	NR	NR	NR	NR	NR	NR	NR	NR	NR	NR	NR	NR	CLP (75 mg), ASP (325 mg)	Complete or near‐complete occlusion	17 (DSA or MRA)	None	None
8	Albuquerque et al., 2011^9^	65/M	R ICA terminus	Unruptured	NR	NR	NR	NR	NR	NR	NR	NR	NR	NR	NR	NR	CLP (75 mg), ASP (325 mg)	Complete or near‐complete occlusion	7 (DSA or MRA)	None	None
9	Albuquerque et al., 2011^9^	38/M	L ICA terminus	Unruptured	NR	NR	NR	NR	NR	NR	NR	NR	NR	NR	NR	NR	CLP (75 mg), ASP (325 mg)	Complete or near‐complete occlusion	NR	NR	None
10	Ahmed et al., 2014^32^	65/F	R ICA terminus	Ruptured	NR	2 × 1.5	2	2	1	1.3	NR	NR	0.9	Prowler Select Plus	Enterprise (double telescopic), no coils	NR	CLP, ASP	NR	24 (NR)	None	None
11	Kim et al., 2015^28^	51/F	R PcomA (incorporating fetal PCA)	Recurrence (post‐coil embolization), unruptured	Severe headache	NR	NR	NR	NR	NR	NR	NR	NR	Prowler Select Plus	Enterprise	4.5 × 22	CLP (75 mg), ASP (100 mg)	Complete occlusion	6 (DSA)	None	None
12	Kitahara et al., 2017^33^	53/F	R ICA terminus	Unruptured	Asymptomatic	12	NR	6	NR	NR	NR	NR	1.4	Prowler Select Plus	Enterprise	4.5 × 14	CLP (75 mg), ASP (100 mg)	Complete occlusion	12 (DSA)	None	None
13	Kwon et al., 2019^10^	50/F	L PcomA (incorporating fetal PCA)	Unruptured	NR	5.7	NR	5.1	0.7	NR	NR	50	1.7	Headway 17	LVIS Jr	3.5 × 23	NR	Near‐complete occlusion	NR	None	None
14	Kwon et al., 2019^10^	80/F	L PcomA (incorporating fetal PCA)	Unruptured	NR	6.3	NR	4.6	0.6	NR	NR	53	1.9	NR	Solitaire	4.0 × 15	NR	Near‐complete occlusion	18 (DSA)	None	None
15	Present case	80/F	R PcomA (incorporating fetal PCA)	Recurrence (post‐clipping), ruptured	Severe headache	2.7 × 2.5 × 1.5	2.7	2.4	1.1	3.1	1.5	40	1.1	SL 10	Neuroform Atlas	4.0 × 21	CLZ (200 mg), ASP (100 mg)	Complete occlusion	24 (DSA)	None	Cerebral infarction (Heubner artery area)

Abbreviations: ASP, aspirin; BA, basilar artery; CLP, clopidogrel; CLZ, cilostazol; DSA, digital subtraction angiography; F, female; ICA, internal carotid artery; L, left; M, male; MRA, magnetic resonance angiography; NR, not reported; PCA, posterior cerebral artery; PcomA, posterior communicating artery; R, right.

The angle of bifurcation between the ICA and PcomA is a crucial factor in successful stent deployment. The bifurcated vessel angle toward the anterograde flow between the afferent and efferent arteries where the stent is placed might be important for efficient and safe catheter delivery. The angle of bifurcation between the ICA and PcomA was acute in all cases (mean, 48°; range, 40°–53°) (Table 1). The angle was the steepest in the present case, at 40°. Caliber of the AcomA is another important factor for the retrograde approach. To ensure success of horizontal stenting via the retrograde route, the caliber of the AcomA should be examined in advance. The minimum size of the AcomA was 0.9 mm (range, 0.9–1.9 mm) in the cases we identified (Table 1). A Prowler Select Plus (Codman Neuroendovascular, Johnson & Johnson) was used as a stent delivery catheter in most cases, with a smaller‐diameter (outer diameter: 1.7 F) catheter (Headway 17; MicroVention and Excelsior SL‐10) used in only two cases (Table 1). Kim et al. pointed out that a communicating artery diameter >1 mm is needed to navigate the stent delivery catheter.^28^ In the present case, AcomA diameter was 1.1 mm on CTA, consequently the microcatheter was easily delivered to the PcomA. Note that a careful procedure is needed to prevent guidewires and microcatheters from migrating into perforating arteries, including the artery of Heubner, which runs parallel to A1. At that time, an accurate roadmap is essential to avoid complications when coil embolization is performed via a retrograde route from the AcomA. As indicated above, although the technique has such risks and complications must be carefully monitored, the approach might be worth considering as an "alternative" option for difficult‐to‐treat patients. However, the indication for the procedure must be carefully selected.

## 4 CONCLUSION

Horizontal stenting via retrograde route might be worth considering as an "alternative" option for coil embolization of ruptured recurrent aneurysms incorporating a fetal variant posterior cerebral artery branching acutely from the internal carotid artery. However, when this method is applied, extreme care should be taken to avoid complications.

## AUTHOR CONTRIBUTIONS

MF, TT, TI, YH, and YM created the research question for this paper. RT, RN, and AT took part in the data collection process. MF wrote the final document. All authors gave substantial contributions to the conception or design of the work, drafted it, and critically revised it for important intellectual content. All authors reviewed and approved the final manuscript.

## CONFLICTS OF INTEREST

The authors report no conflict of interest.

## ETHICAL APPROVAL

Not applicable.

## CONSENT

Written informed consent was obtained from the patient to publish this report in accordance with the journal's patient consent policy.

## Data Availability

None.
